# GPX8 as a Novel Prognostic Factor and Potential Therapeutic Target in Primary Glioma

**DOI:** 10.1155/2022/8025055

**Published:** 2022-08-23

**Authors:** Zhao-shou Yang, Qin Yang, Xiao-xiao Sun, Kui Xiong, Xiao-ting Zhu, Yi-chen Wang, Qian-yao Ren, Guo-hui Wu, Shi-min Wang, Xu-qin Cao, Xiao-rong Yang, Wen-gong Jiang

**Affiliations:** ^1^The First Affiliated Hospital/School of Clinical Medicine of Guangdong Pharmaceutical University, Guangdong Pharmaceutical University, Guangzhou, Guangdong 510080, China; ^2^Zhongshan School of Medicine, Sun Yat-sen University, Guangzhou 510080, China; ^3^Department of Ultrasound and Electrocardiogram, Sun Yat-sen University Cancer Center, State Key Laboratory of Oncology in South China, and Collaborative Innovation Center for Cancer Medicine, Guangzhou 510080, China

## Abstract

One of the most prevalent malignant primary brain tumors is primary glioma. Although glutathione peroxidase 8 (GPX8) is intimately associated with carcinogenesis, its function in primary gliomas has not yet been thoroughly understood. Here, we leveraged Chinese Glioma Genome Atlas (CGGA), The Cancer Genome Atlas (TCGA), and Genotype-Tissue Expression (GTEx) database to investigate the association between GPX8 and overall survival (OS) of patients with primary gliomas, and our results showed that GPX8 expression was negatively correlated with OS. Moreover, the expression of GPX8 is significantly lower in normal tissue when compared to glioma tissue. According to results of univariate and multivariate analysis from CGGA using R studio, GPX8 is a valuable primary glioma prognostic indicator. Interestingly, high GPX8 expression is correlated positively with the hedgehog and kras signaling pathways and negatively with G2 checkpoint, apoptosis, reactive oxygen species (ROS) pathway, and interferon gamma pathway, which could be beneficial for the proliferation of glioma cells. Furthermore, GPX8 knockdown caused G1 cell cycle arrest, increased cell death, and reduced colony formation in U87MG and U118MG cells. In conclusion, GPX8 is a promising therapeutic target and meaningful prognostic biomarker of primary glioma.

## 1. Introduction

The majority of primary central nervous system tumors, approximately 50–60% of all malignant brain tumors, are gliomas [1, 2]. The World Health Organization (WHO) has categorized gliomas into grades I through IV based on histology and molecular genetic abnormalities [2, 3]. Glioma is conventionally treated with surgery followed by chemo- and radiotherapies [4]. Despite active treatments used today, overall survival (OS) and progression-free survival in patients with glioma remain predictable [5, 6]. Therefore, it is urgently required to search for novel and valuable glioma prognostic or predictive biomarkers, which could notably increase effectiveness for glioma therapy [2, 6]. Numerous studies have shown that mRNA is involved in regulating glioma pathology, and several mRNAs have been found to be promising biomarkers to predict the OS time of glioma patients [7, 8].

The glutathione peroxidase family (GPXs) consists of 8 enzymes, which can eliminate H_2_O_2_ and prevent lipotoxicity [9]. Some of them have been discovered to be strongly related to carcinogenesis, although their main protective effect is in decreasing damage caused by intracellular oxidative stress [10]. GPX8, as the last discovered member of the GPXs, is a 209-amino acid-long type II transmembrane protein located in the endoplasmic reticulum. It contains an N-terminal signal peptide and a C-terminal endoplasmic reticulum (ER) membrane localization signal and functions in regulating calcium homeostasis [11]. In addition, lots of studies have shown that GPX8 is linked to various sorts of human malignant tumors [12–15].

Previously, several research groups explored the potential role of GPX8 in glioma patients, but their studies were only based on The Cancer Genome Atlas (TCGA) [14, 15]. Here, we further investigated this gene by combined using the Chinese Glioma Genome Atlas (CGGA) databases and a variety of algorithms. Our multivariate analysis showed that GPX8 was not a good prognostic indicator for the OS of all types of gliomas. Instead, it is a valuable prognostic biomarker specific to primary glioma. Moreover, we firstly found that knocking down GPX8 expression in U87MG and U118MG cells led to cell cycle arrest, increased cell apoptosis, and decreased colony formation capacity. Hence, we concluded that GPX8 is a meaningful predictive biomarker for primary glioma and a promising antiglioma therapeutic target.

## 2. Materials and Methods

### 2.1. Data Source

The clinical information and glioma RNA sequencing data were extracted from the CGGA Data Portal (http://www.cgga.org.cn/) and TCGA Data Portal (https://tcga-data.nci.nih.gov/tcga/). The gene expression of normal brain tissue was extracted from the Genotype-Tissue Expression (GTEx) Data Portal (https://xenabrowser.net/datapages/) [16]. We downloaded clinical and RNA expression data of glioma patients from TCGA and glioma patients (DataSet ID: mRNAseq_693 and mRNAseq_325) from CGGA. The sva package and limma package in R language were applied to combine gene expression and clinical data for subsequent analysis. And 207 normal brain tissue (Brain-Frontal Cortex and Brain-Cortex) mRNA expression data were downloaded from GTEx.

### 2.2. Survival and ROC Curve Analysis

The correlation between GPX8 expression and OS were analysed with the Kaplan-Meier Plotter (https://kmplot.com/analysis/). Diagnosis and prognosis were determined with the calculation of the area under the ROC curves.

### 2.3. Gene Set Enrichment Analysis (GSEA)

GSEA is used to analyse the signal pathways and functions of genes. The primary glioma samples from CGGA were divided into high GPX8-expression group and low GPX8-expression group. Nominal *P* values and normalized enrichment scores (NES) were used to identify and sort enriched pathways. The difference is significant when *P* value is less than 0.05 and the FDR value is less than 0.25.

### 2.4. Cell Culture and Transfection

U87MG and U118MG cell lines were bought from Procell (Wuhan, China). Cells were grown in DMEM medium with 10% FBS under ideal circumstances (37°C; 5% CO_2_). siRNAs were obtained from Tsingke Biotechnology Co., Ltd. (Beijing, China). The sequences of siRNAs for GPX 8 are 5′-GGUCAAGUUGUGAAGUUCUTT-3′ and 5′-AGAACUUCACAACUUGACCTT-3′. All siRNAs were transfected to cells by RNAi-Mate (Genepharma, Shanghai, China).

### 2.5. Western Blot Assay (WB)

Cells were lysed in RIPA buffer (Fude, Hangzhou, China). Cell lysates were mixed with protein loading buffer and boiled for 5 minutes to make protein samples. Protein samples were separated with a sodium dodecyl sulfate-polyacrylamide gel electrophoresis gel and transferred to the polyvinylidene fluoride membrane (PVDF). With the use of a fast-blocking buffer (Beyotime, Shanghai, China), the membrane containing proteins was blocked and then incubated at 4°C overnight with primary antibodies (GPX8, Abclonal, China; Actin, Beyotime, China). Horseradish peroxidase- (HRP-) conjugated antibodies are secondary antibodies. After washing, the substrate of HRP (Fude, Hangzhou, China) was applied to detect the target protein.

### 2.6. Cell Viability

Cell Counting Kit-8 (CCK-8) (Topscience, Shanghai, China) was applied to measure cell viability. Exponentially growing cells were seeded into 96-well plates at a concentration of 5000 cells per well. 36 hours after seeding, CCK-8 solution was added and incubated at 37°C for four hours. Then at 450 nm, the optical density (OD) value was measured.

### 2.7. Colony Formation Assay

36 hours after siRNA transfection, cells were seeded at a density of 500 cells per well in six-well plates with 2 mL of DMEM medium containing 10% FBS. Two weeks after incubation at 37°C, the culture media was removed and the cells in plates were fixed with ice-cold methanol and stained with crystal violet (Solarbio, China) for twenty minutes. The numbers of colonies were counted after staining.

### 2.8. Cell Apoptosis and Cycle Assay

36 hours after siRNA transfection, cells were plated into 24-well plates at the concentration of 50000 cells per well. 24 hours after seeding, cells were collected and divided into two parts. One part was used for cell apoptosis assay. Briefly, cells were stained with propidium iodide (PI) and Annexin V-FITC following the manufacturer's instructions (KeyGEN, Jiangsu, China). Flow cytometer was used to detect cell apoptosis. Another part was used for cell cycle assay (Beyotime, Shanghai, China). Collecting cells were rinsed in ice-cold PBS before being fixed in 75% methanol over the night at 4°C. Following that, cells were stained for 20 minutes with PI, RNase A, and Triton X-100. Cell apoptosis assay and cell cycle assay were performed using flow cytometry.

### 2.9. Wound Healing Assay

U87MG and U118MG cells were cultured in 12-well plates to reach 100% confluence. Wounds were made using sterilized and clean pipette tips. Media were replaced with DMEM medium with 1% FBS. After replacing the media, digital pictures were obtained using an inverted microscope at 0, 12, and 24 hours. A relative migration rate was calculated by normalizing the distance of the wound area at 0 hour.

### 2.10. Statistical Analysis

The gene expression data from the public database were analysed using R (v.4.0.5) to get all statistical testing and graphing. All *P* values were two-sided, and significance was defined as a value below 0.05. The comparison of GPX8 expression between nonpaired samples was evaluated using the Wilcoxon rank test. The correlation between GPX8 expression levels and clinicopathological factors was examined using logistic regression analysis, Kruskal Wallis, and Wilcoxon symbolic rank tests. Survival curves plotted using the Kaplan–Meier method were compared to the log-rank test to explore the prognostic value of GPX8 in primary glioma patients.

## 3. Results

### 3.1. GPX8 Was Highly Expressed in Glioma

**Figure 1:**
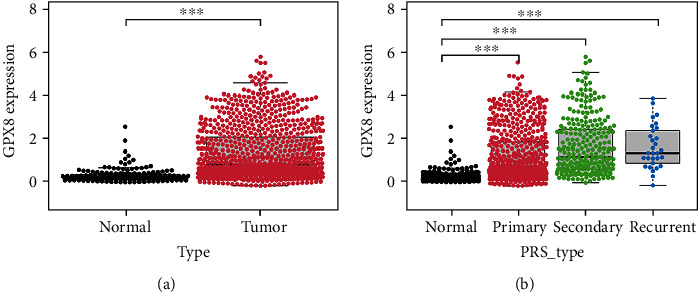
GPX8 expression in glioma and normal brain tissues. (a) GPX8 was highly expressed in gliomas tissues (*n* = 925) from CGGA compared to normal brain tissues (*n* = 207) from GTEx. (b) GPX8 expression was increased in primary (*n* = 620), secondary (*n* = 275), and recurrent (*n* = 30) gliomas. ^∗∗∗^*P* < 0.001.

RNA-sequencing data of WHO II-IV gliomas and normal brain tissues were downloaded from CGGA, TCGA, and GTEx. Data analysis was done by limma and sva package in R studio. The result revealed that GPX8 was highly expressed in gliomas (see Figure 1(a) and Figure S1 in the Supplementary Materials). Then, we further analysed the data of primary gliomas after excluding recurrent and secondary gliomas from CGGA [17]. The characteristics of patients with glioma are summarized in Table 1. Further analysis indicated that GPX was also highly expressed in primary gliomas (Figure 1(b)). In brief, GPX8 is highly expressed in gliomas tissue, including primary, recurrent, and secondary gliomas.

### 3.2. GPX8 Is a Potential Prognostic Biomarker in Gliomas

**Figure 2:**
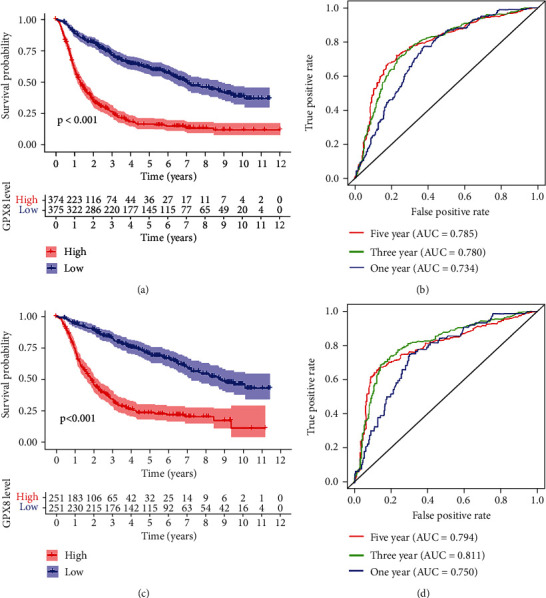
The survival curves and the ROC curves. (a) High GPX8 expression in all types of gliomas was associated with shorter overall survival. High : low = 374 : 375; HR 1.4-1.622; *P* < 0.001. (b) ROC curves showed that GPX8 as a prognostic marker was valuable for all types of gliomas. (c) High GPX8 expression in primary gliomas was associated with shorter overall survival. High : low = 251 : 251; HR 1.6-1.847; *P* < 0.001. (d) ROC curves showed that GPX8 had a medium diagnostic accuracy in primary gliomas.

The results of the survival analysis using the CGGA databases revealed that GPX8 expression significantly affects prognosis in gliomas, including primary glioma (Figure 2). Moreover, the diagnostic value of GPX8 level in glioma was evaluated by exploring the receiver operating characteristic (ROC) curve. As demonstrated in Figures 2(b) and 2(d), GPX8 had a medium diagnostic accuracy in gliomas (AUCs were above 0.7 and even 0.8). In short, results above supported that GPX8 is a potential diagnostic biomarker in glioma, including primary gliomas.

### 3.3. Correlation between GPX8 Expression and Clinical Features

**Figure 3:**
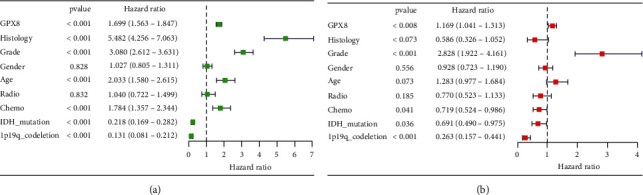
Relationship between clinical characteristics and prognosis of patients with primary glioma (*n* = 502) from CGGA database. (a) Univariate analysis. (b) Multivariate analysis.

**Figure 4:**
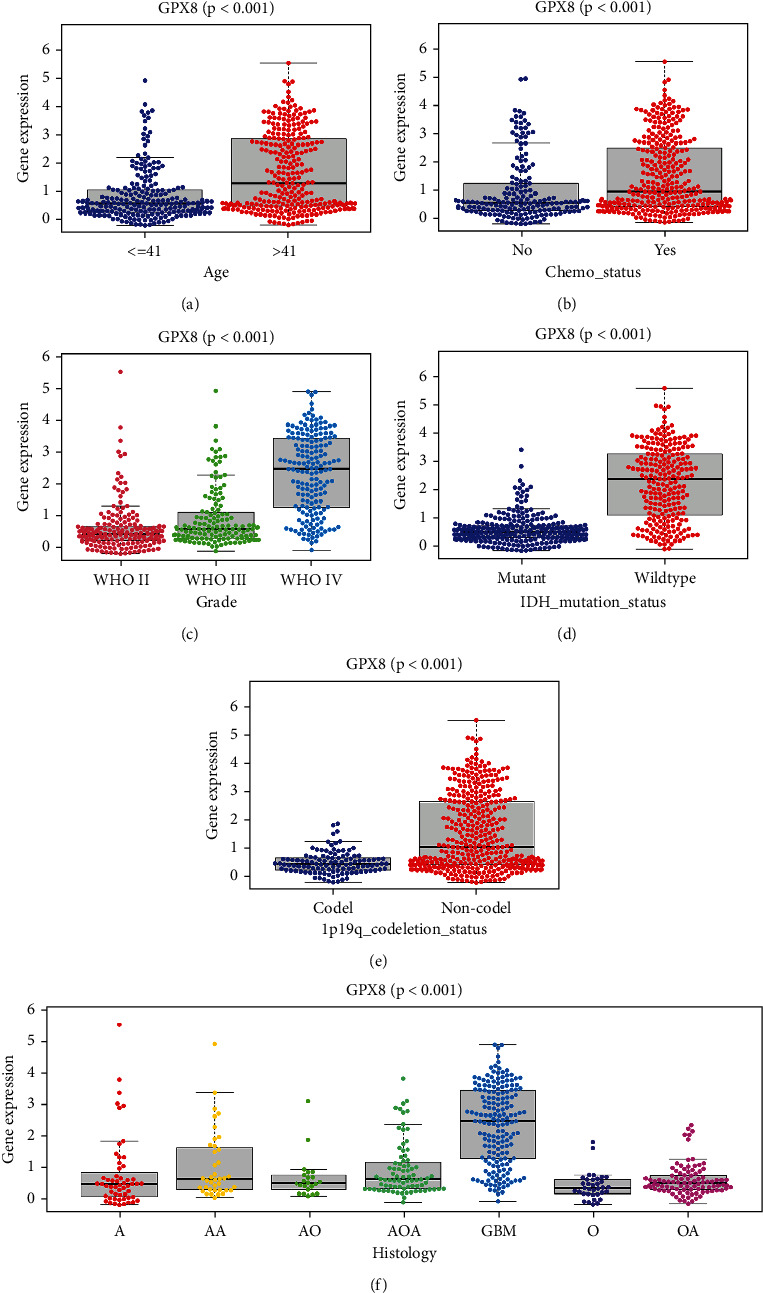
Relationship between GPX8 gene expression and clinical features of primary gliomas (*n* = 502).

Though difference of RNA expression, survival, and ROC curve has been explored, further analysis is needed to do to confirm the potential role of GPX8 in gliomas. With clinical information from CGGA, univariate and multivariate analysis was done in this study (see Figure 3 and Figure S2 in the Supplementary Materials). Surprisingly, the correlation between GPX8 expression and OS was not significant in mixed glioma type analysis (univariate analysis, *P* < 0.001, HR 1.431-1.622; multivariate analysis, *P* = 0.074, HR 0.991-1.174, Figure S2). Interestingly, subgroup analysis showed that this correlation was significant only in the primary glioma subgroup (univariate analysis, *P* < 0.001, HR 1.563-1.847; multivariate analysis, *P* = 0.008, HR 1.041-1.313) (Figure 3). So, next we focused on analysing the correlation of GPX8 with clinical characteristics of primary gliomas. In primary gliomas, the GPX8 was associated with age, WHO grade, chemo, IDH mutation, histology, and 1p19q_codeletion (Figure 4).

### 3.4. Enrichment Analysis of GPX8 Gene Functional Networks

**Figure 5:**
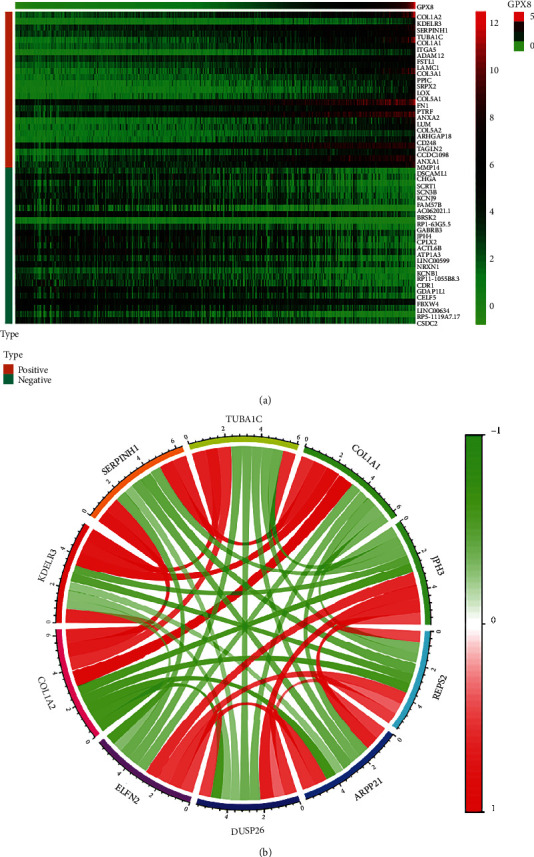
Coexpression analysis. (a) Gene expression heat map and correlations for GPX8 coexpressed genes. (b) The coexpressed network of 5 positively related and 5 negatively related genes for GPX8.

Pearson correlation analysis was further applied to identify GPX8-related genes to elucidate the role of GPX8 in the pathophysiology of gliomas. The top 25 significant genes that correlated positively or negatively with GPX8 were shown in the heat maps (Figure 5(a)), which suggested a widespread impact of GPX8 in the transcription. Top five positively correlated genes (COL1A2, KDELR3, SERPINH1, TUBA1C, and COL1A1) and top five negatively correlated genes (JPH3, REPS2, ARPP21, DUSP26, and ELFN2) (Table 2) were selected to draw a coexpressed network, as shown in Figure 5(b).

**Figure 6:**
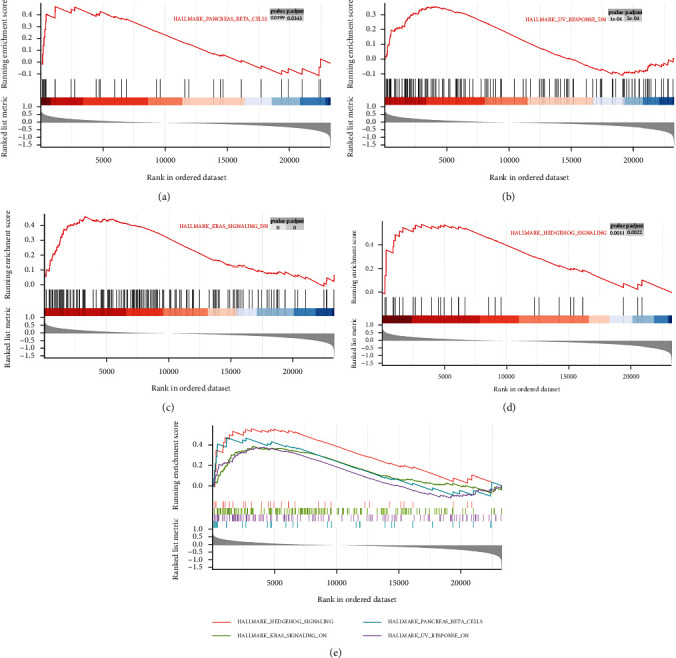
High GPX8 expression is significantly correlated with (a) pancreas beta pathway, (b) UV response, (c) kras signaling, (d) hedgehog signaling pathway, and (e) merged four pathways.

**Figure 7:**
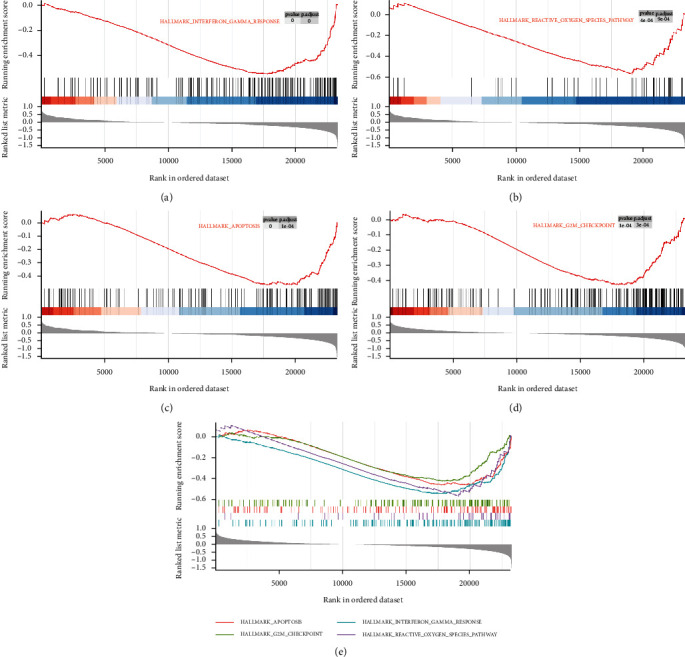
Low GPX8 expression is significantly correlated with (a) interferon gamma response, (b) reactive oxygen species pathway, (c) apoptosis, (d) G2/M checkpoints, and (e) merged four pathways.

We next explored signaling pathways differentially expressed in patients with primary glioma as a function of GPX8 expression via GSEA. Table S1 in the Supplementary Materials lists out all significantly enriched signaling pathways based upon NES values. As shown in Figure 6, hedgehog (HH) signaling pathway, kras pathway, pancreas beta cells, and UV response were significantly associated with high GPX8 expression in patients with primary glioma; in contrast, interferon gamma response, G2/M checkpoint, apoptosis, and reactive oxygen species (ROS) pathway with low GPX8 expression (Figure 7). Interestingly, these enriched pathways are closely related to carcinogenesis.

### 3.5. Validation of GPX8 Function in Gliomas *In Vitro*

**Figure 8:**
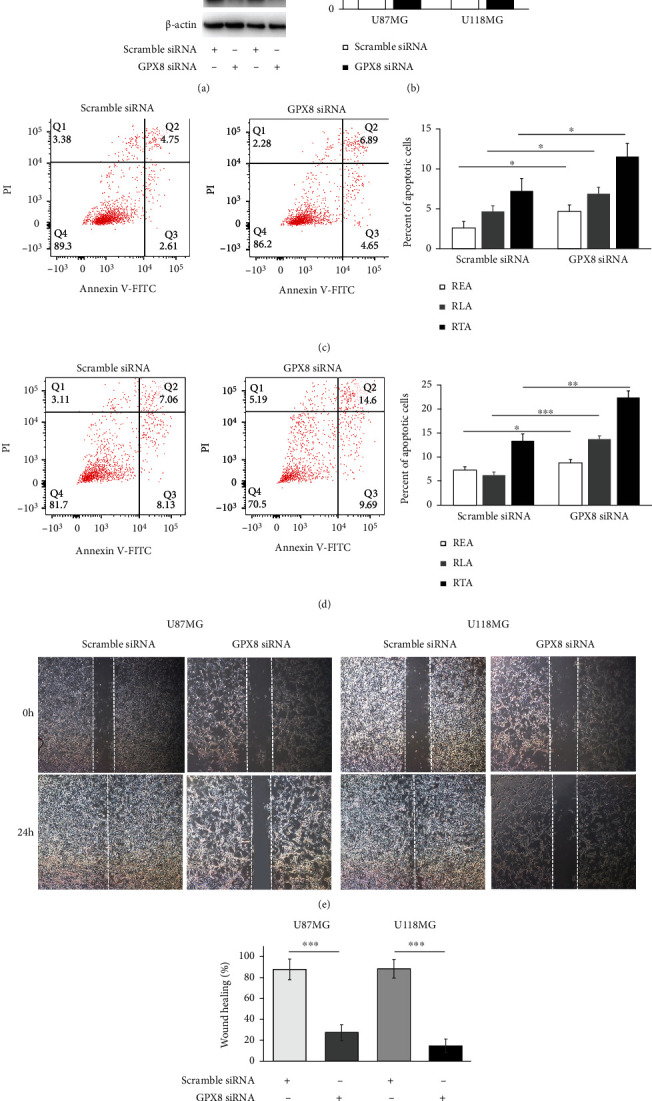
Effects of GPX8 on cell proliferation, apoptosis, and migration. (a) The decreased protein level of GPX8 in U87MG and U118 cell lines was proved by western blotting. (b) Cell proliferation assay (^∗∗∗^*P* < 0.001). (c, d) Apoptosis assay (^∗^*P* < 0.05, ^∗∗^*P* < 0.01, and ^∗∗∗^*P* < 0.001). REA: the rate of early apoptotic cells; RTA: the rate of total apoptotic cells; RLA: the rate of late apoptotic cells. (e, f) Wound healing assay (^∗∗∗^*P* < 0.001).

**Figure 9:**
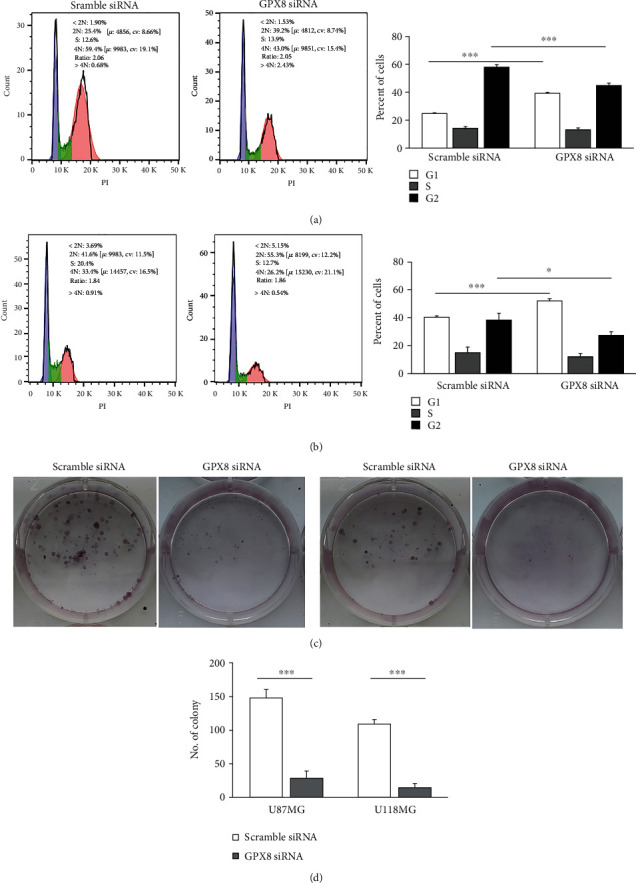
Effects of GPX8 on cell cycle and colony formation capacity. (a, b) Cell cycle assay (^∗^*P* < 0.05, ^∗∗∗^*P* < 0.001). G1: 2N; G2: 4N. (c, d) Colony formation capacity assay (^∗∗∗^*P* < 0.001).

*In vitro* experiments were performed to validate the GPX8 function in gliomas. The GPX8 expression in two glioma cell lines (U87MG and U118MG) was successfully knocked down by transfection of GPX8 siRNA, which was confirmed by WB (Figure 8(a)). Comparing with scramble control, GPX8 knockdown contributed to the inhibition of cell proliferation (Figure 8(b)), the increase of apoptotic cells (Figures 8(c) and 8(d)), and the decrease of wound healing rate (Figures 8(e) and 8(f)). Meanwhile, inhibition of GPX8 expression resulted in cell cycle arrest at the G1 (2N) phase and weakened colony formation capacity (Figure 9). Above data suggested GPX8 plays an important role in the proliferation, cell cycle, apoptosis, migration, and colony formation capacity of glioma cells, which would be beneficial for disease development.

## 4. Discussion

One of the most common malignant brain tumor in adults is primary glioma [2]. Searching for new tumor biomarkers is beneficial for the efficiency of glioma therapy [2]. Though major favorable prognostic biomarkers have been reported, novel biomarkers are continuously being explored. And bioinformatics analysis is a powerful strategy to facilitate discovery of new biomarkers. Hence, this study was performed to look for novel prognostic factors.

Several novel prognostic and therapy targeted factors in gliomas have been found based on public database [4, 8, 14, 15, 18, 19]. For example, GPX1 expression was a potential prognostic factor in low-grade glioma (LGG) and so was GPX8 in GBM/LGG, based on bioinformatic analysis from TCGA [14, 15, 18]. GPX7 is closely related to the malignant clinical features of gliomas from CCGA [4]. While glioma patients in TCGA are from western countries, CGGA is another important public database which collected RNA expression and clinical information of Chinese glioma patients. And race and ethnicity could be important factors that may affect prognosis in gliomas [20, 21]. Hence, these prognostic genes proved based on TCGA are needed to reconfirm in CGGA database. Surprisingly, although GPX8 is proved to be a potential prognostic factor in glioma based on analysis of data from TCGA [14, 15], our analysis results suggested that GPX8 is not a reliable prognostic factor in the analysis of mixed-type gliomas from CGGA (Figure S2). Further, by excluding recurrent and secondary gliomas, we solely analysed the correlation between GPX8 expression and the primary gliomas from CGGA by bioinformatics tools. Our results favored that GPX8 is a promising prognostic factor mainly in primary glioma rather than in all types of gliomas. And GPX8 expression is significantly correlated with grade, IDH1/2 mutation, and 1p/19q codeletion in primary glioma from CGGA, which are favorable prognostic factors in glioma.

In addition, coexpressed genes of GPX8 in primary gliomas from CGGA were analysed. The top positively and negatively correlated genes are displayed in Figure 5. Among them, ATP1A3, COL1A1, COL1A2, LOX, FN1, and ANXA1 are involved in ferroptosis [22–26], which hints that GPX8 could be a cofactor in ferroptosis. TUBA1C, which is positively correlated with GPX8, has been reported to be a prognostic marker in LGG and correlated with immune cell infiltration in the tumor microenvironment [27]. DUSP26 is negatively correlated with GPX8, and low DUSP26 expression could lead to malignant behavior in glioblastoma cells by deregulating MAPK and Akt signaling pathway [28]. NDRG2 expressed in astrocytes of the central nervous system is negatively correlated with gpx8, which is involved in cell proliferation and differentiation and generally considered as a tumor suppressor [29]. GPX8 could exert its carcinogenesis function in primary glioma accompanying the dysfunction of these coexpressed genes.

In the present study, gene set enrichment analysis of GPX8 in primary glioma was performed (Figures 6 and 7). These enriched pathways play important roles in human cancers. The HH signaling pathway regulates cell growth and differentiation, but its dysfunction could promote the development of human cancers including gliomas [30, 31]. Kras is the most frequent oncogenic gene involved in many cancers [32]. Interferon *γ* (IFN-*γ*) has antitumor effect through modulating the immunity within the tumor microenvironment [33]. Low levels of ROS help glioma cells resist therapy [34]. Low expression of GPX8 is positively correlated with apoptosis and G2M checkpoint (Figure 7). Lots of anticancer drugs exert their function by inducing cell apoptosis and G2M checkpoint [35–37]. Meanwhile, GPX8 function in gliomas was validated by *in vitro* experiments (Figures 8 and 9). Some research groups have proved that GPX8 knockdown results in the suppression of the proliferation, colony formation capacity, and migration of glioma cells [14, 15]. But they only used one glioma cell line and did not fully study the role of GPX8 expression in glioma cell apoptosis and cycle. In the present study, we firstly reported that decreased GPX8 expression led to increased cell apoptosis and cell arrest at the G1 stage using two glioma cell lines.

## 5. Conclusions

In conclusion, the present study found that GPX8 is a favorable prognostic factor in primary glioma. And the intervention of GPX8 in glioma tissue could be a promising therapy treatment for primary glioma treatments and other brain-related tumors.

## Figures and Tables

**Table 1:** Characteristics of patients with primary glioma based on CGGA.

Characteristics		Number of cases	Percentages (%)
Gender	Male	294	58.5
	Female	208	41.5
Age	≤41	223	45.79
	>41	279	54.21
Histology	Astrocytoma	55	10.9
	Anaplastic astrocytoma	39	7.7
	Anaplastic oligodendroglioma	22	4.3
	Anaplastic oligoastrocytoma	80	10.68
	Glioblastoma	176	35
	Oligodendroglioma	35	6.9
	Oligoastrocytoma	95	15.9
IDH_mutation_status	Mutant	270	53.7
	Wild type	232	46.3
1p19q_codeletion_status	Noncodel	389	22.6
	Codel	113	77.4
Radio_status	Yes	426	84.8
	No	76	15.2
Chemo_status	Yes	235	64.7
	No	177	35.3
Grade	WHO II	185	36.8
	WHO III	141	35
	WHO IV	176	28

**Table 2:** Description of ten coexpressed genes showed in Figure 5(b).

Gene	Cor.	*P* value
GPX8	1	0
COL1A2	0.845	6.89*E* − 279
KDELR3	0.844	4.46*E* − 277
SERPINH1	0.841	7.55*E* − 273
TUBA1C	0.834	4.98*E* − 265
COL1A1	0.828	5.27*E* − 257
JPH3	-0.609	2.37*E* − 104
REPS2	-0.611	2.88*E* − 105
ARPP21	-0.614	1.92*E* − 106
DUSP26	-0.625	1.47*E* − 111
ELFN2	-0.643	7.22*E* − 120

## Data Availability

The data used to support the findings of this study are included within the article.

## Supplementary Materials

Supplementary Materials Figure S1: comparison of GPX8 expression between all glioma tissues (*n* = 670) from TCGA and normal brain tissues (*n* = 207) from GTEx. ^∗∗∗^*P* < 0.001. Figure S2: relationship between clinical characteristics and prognosis of patients (*n* = 925) with all types of gliomas (*n* = 749) from CGGA database. (a) Univariate analysis. (b) Multivariate analysis. Table S1: enriched pathways via GSEA.
