# Supplementary material for: GPX8 as a Novel Prognostic Factor and Potential Therapeutic Target in Primary Glioma
Source: J Immunol Res. 2022 Aug 23;2022:8025055. doi: 10.1155/2022/8025055 (PMC9427289; doi:10.1155/2022/8025055)
Supplement: Supplementary Materials — Figure S1: comparison of GPX8 expression between all glioma tissues (n = 670) from TCGA and normal brain tissues (n = 207) from GTEx. ∗∗∗P < 0.001. Figure S2: relationship between clinical characteristics and prognosis of patients (n = 925) with all types of gliomas (n = 749) from CGGA database. (a) Univariate analysis. (b) Multivariate analysis. Table S1: enriched pathways via GSEA. [file 8025055.f1.docx]

## Supplementary Materials


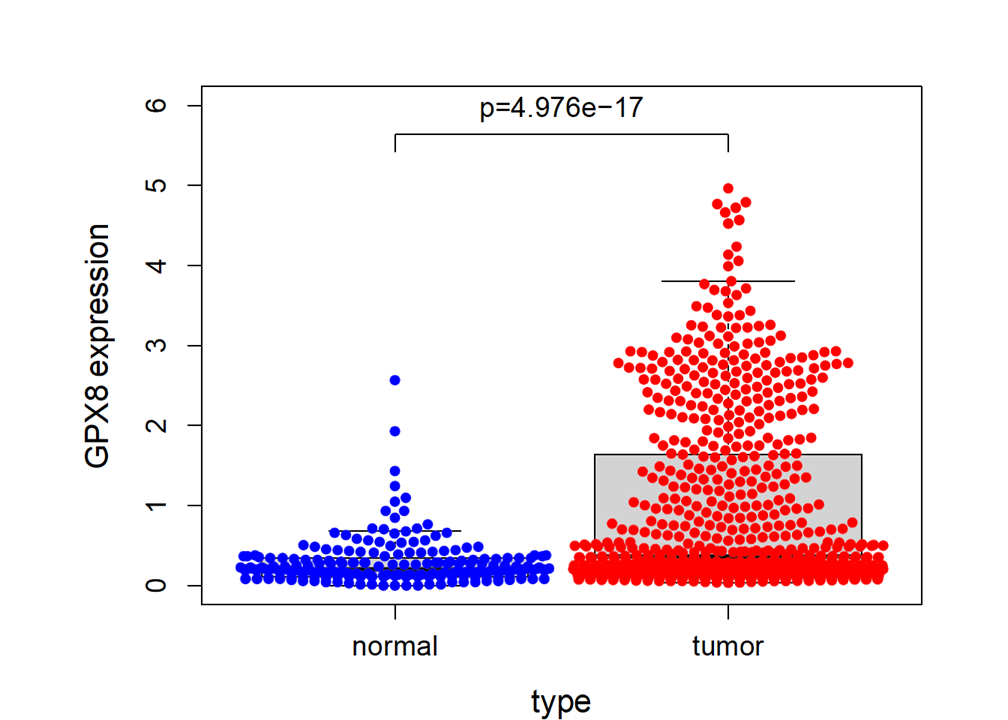


Figure S1: Comparison GPX8 expression between all glioma tissues (n = 670) from TCGA and normal brain tissues (n=207) from GTEx. ***P < 0.001.


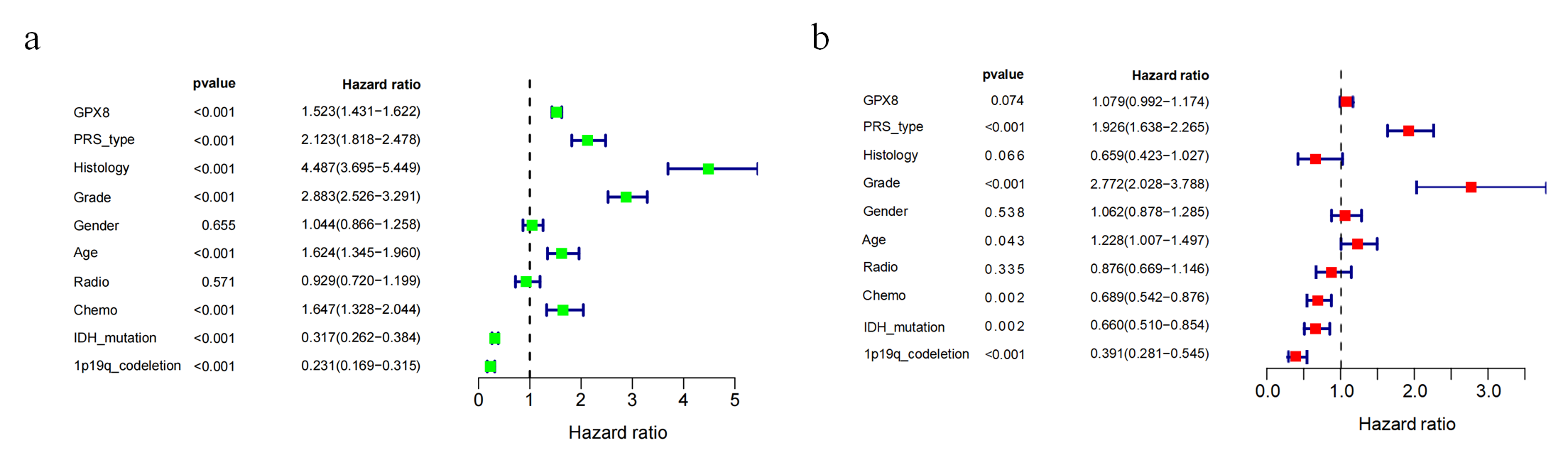


Figure S2: Relationship between clinical characteristics and prognosis of patients (n = 925) with all types of gliomas (n = 749) from CGGA database. (a) Univariate analysis. (b) Multivariate analysis.

Table S1: Enriched pathways via GSEA

| ID | Description | setSize | enrichmentScore | NES | pvalue | p.adjust | qvalues |
| --- | --- | --- | --- | --- | --- | --- | --- |
| HALLMARK_MYC_TARGETS_V1 | HALLMARK_MYC_TARGETS_V1 | 192 | -0.62545 | -2.48681 | 1.00E-10 | 8.33E-10 | 3.16E-10 |
| HALLMARK_EPITHELIAL_MESENCHYMAL_TRANSITION | HALLMARK_EPITHELIAL_MESENCHYMAL_TRANSITION | 193 | -0.5575 | -2.21654 | 1.00E-10 | 8.33E-10 | 3.16E-10 |
| ALLMARK_DNA_REPAIR | HALLMARK_DNA_REPAIR | 147 | -0.56497 | -2.18533 | 1.00E-10 | 8.33E-10 | 3.16E-10 |
| HALLMARK_COAGULATION | HALLMARK_COAGULATION | 117 | -0.57838 | -2.18291 | 3.07E-10 | 1.92E-09 | 7.27E-10 |
| HALLMARK_OXIDATIVE_PHOSPHORYLATION | HALLMARK_OXIDATIVE_PHOSPHORYLATION | 180 | -0.53451 | -2.11295 | 1.00E-10 | 8.33E-10 | 3.16E-10 |
| HALLMARK_INTERFERON_GAMMA_RESPONSE | HALLMARK_INTERFERON_GAMMA_RESPONSE | 189 | -0.53136 | -2.10989 | 1.00E-10 | 8.33E-10 | 3.16E-10 |
| HALLMARK_ALLOGRAFT_REJECTION | HALLMARK_ALLOGRAFT_REJECTION | 173 | -0.5288 | -2.08503 | 1.52E-10 | 1.09E-09 | 4.12E-10 |
| HALLMARK_E2F_TARGETS | HALLMARK_E2F_TARGETS | 194 | -0.52345 | -2.08319 | 1.00E-10 | 8.33E-10 | 3.16E-10 |
| HALLMARK_INTERFERON_ALPHA_RESPONSE | HALLMARK_INTERFERON_ALPHA_RESPONSE | 94 | -0.56356 | -2.07398 | 1.03E-07 | 5.74E-07 | 2.18E-07 |
| HALLMARK_MYC_TARGETS_V2 | HALLMARK_MYC_TARGETS_V2 | 58 | -0.56826 | -1.94034 | 2.15E-05 | 8.97E-05 | 3.40E-05 |
| HALLMARK_UNFOLDED_PROTEIN_RESPONSE | HALLMARK_UNFOLDED_PROTEIN_RESPONSE | 106 | -0.5165 | -1.92221 | 2.74E-06 | 1.37E-05 | 5.18E-06 |
| HALLMARK_REACTIVE_OXYGEN_SPECIES_PATHWAY | HALLMARK_REACTIVE_OXYGEN_SPECIES_PATHWAY | 46 | -0.55364 | -1.80634 | 0.000615 | 0.001336 | 0.000506 |
| HALLMARK_APOPTOSIS | HALLMARK_APOPTOSIS | 157 | -0.45038 | -1.76075 | 1.25E-05 | 5.70E-05 | 2.16E-05 |
| HALLMARK_IL6_JAK_STAT3_SIGNALING | HALLMARK_IL6_JAK_STAT3_SIGNALING | 81 | -0.48239 | -1.73894 | 0.00049 | 0.001212 | 0.000459 |
| HALLMARK_GLYCOLYSIS | HALLMARK_GLYCOLYSIS | 189 | -0.42383 | -1.68291 | 4.42E-05 | 0.000158 | 5.98E-05 |
| HALLMARK_HYPOXIA | HALLMARK_HYPOXIA | 183 | -0.4215 | -1.66906 | 3.79E-05 | 0.000146 | 5.53E-05 |
| HALLMARK_TNFA_SIGNALING_VIA_NFKB | HALLMARK_TNFA_SIGNALING_VIA_NFKB | 194 | -0.4139 | -1.6472 | 6.52E-05 | 0.000204 | 7.73E-05 |
| HALLMARK_G2M_CHECKPOINT | HALLMARK_G2M_CHECKPOINT | 187 | -0.41155 | -1.63123 | 9.34E-05 | 0.000275 | 0.000104 |
| HALLMARK_P53_PATHWAY | HALLMARK_P53_PATHWAY | 190 | -0.40683 | -1.61592 | 0.000163 | 0.000452 | 0.000171 |
| HALLMARK_COMPLEMENT | HALLMARK_COMPLEMENT | 190 | -0.3984 | -1.58245 | 0.000317 | 0.000834 | 0.000316 |
| HALLMARK_MTORC1_SIGNALING | HALLMARK_MTORC1_SIGNALING | 195 | -0.39273 | -1.56334 | 0.000509 | 0.001212 | 0.000459 |
| HALLMARK_IL2_STAT5_SIGNALING | HALLMARK_IL2_STAT5_SIGNALING | 193 | -0.38527 | -1.53177 | 0.000656 | 0.001367 | 0.000518 |
| HALLMARK_ESTROGEN_RESPONSE_EARLY | HALLMARK_ESTROGEN_RESPONSE_EARLY | 190 | 0.298635 | 1.430187 | 0.001187 | 0.002373 | 0.000899 |
| HALLMARK_ANDROGEN_RESPONSE | HALLMARK_ANDROGEN_RESPONSE | 93 | 0.345335 | 1.452368 | 0.008372 | 0.015503 | 0.005875 |
| HALLMARK_PANCREAS_BETA_CELLS | HALLMARK_PANCREAS_BETA_CELLS | 30 | 0.455287 | 1.536396 | 0.015764 | 0.028151 | 0.010668 |
| HALLMARK_UV_RESPONSE_DN | HALLMARK_UV_RESPONSE_DN | 137 | 0.33975 | 1.549443 | 0.00061 | 0.001336 | 0.000506 |
| HALLMARK_KRAS_SIGNALING_DN | HALLMARK_KRAS_SIGNALING_DN | 157 | 0.353456 | 1.634532 | 6.02E-05 | 0.000201 | 7.61E-05 |
| HALLMARK_HEDGEHOG_SIGNALING | HALLMARK_HEDGEHOG_SIGNALING | 33 | 0.523343 | 1.783341 | 0.001534 | 0.00295 | 0.001118 |
